# Supplementary material for: Complex‐centric proteome profiling by SEC‐SWATH‐MS
Source: Mol Syst Biol. 2019 Jan 14;15(1):e8438. doi: 10.15252/msb.20188438 (PMC6346213; doi:10.15252/msb.20188438)
Supplement: Supplementary file 8 — Dataset EV7 [file MSB-15-e8438-s008.zip › feature_plots_string/Q96SB8.pdf]

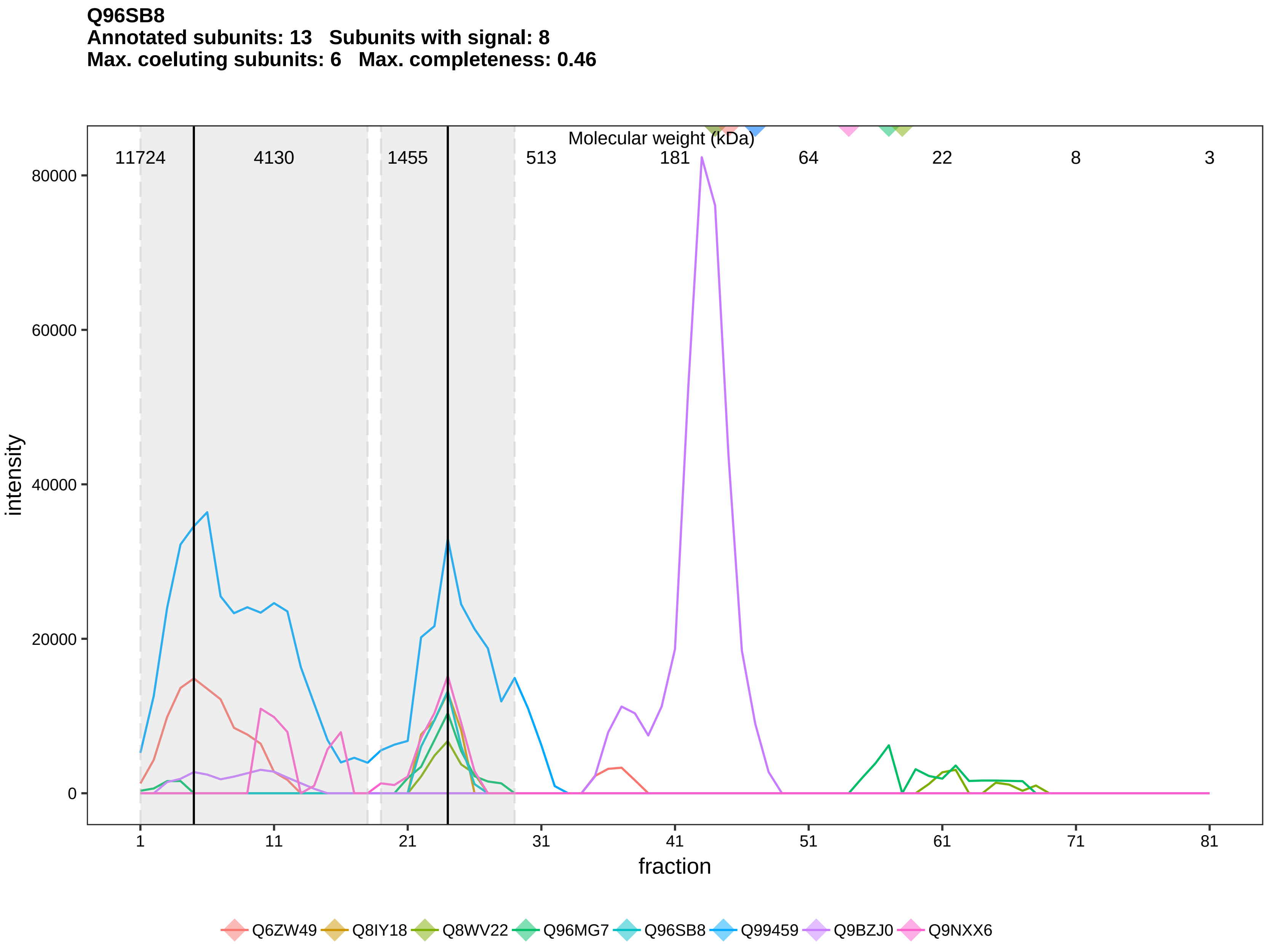

Q96SB8
Annotated subunits: 13 Subunits with signal: 8
Max. coeluting subunits: 6 Max. completeness: 0.46
Molecular weight (kDa)
11724
4130
1455
513
181
64
22
8
3
intensity
80000
60000
40000
20000
0
fraction
1
11
21
31
41
51
61
71
81
Q6ZW49
Q8IY18
Q8WV22
Q96MG7
Q96SB8
Q99459
Q9BZJ0
Q9NXX6
